# Magneto-Thermoelastic Response in an Unbounded Medium Containing a Spherical Hole via Multi-Time-Derivative Thermoelasticity Theories

**DOI:** 10.3390/ma15072432

**Published:** 2022-03-25

**Authors:** Ashraf M. Zenkour, Daoud S. Mashat, Ashraf M. Allehaibi

**Affiliations:** 1 Department of Mathematics, Faculty of Science, King Abdulaziz University, P.O. Box 80203, Jeddah 21589, Saudi Arabia; dmashat@kau.edu.sa (D.S.M.); amlehaibi@uqu.edu.sa (A.M.A.); 2 Department of Mathematics, Faculty of Science, Kafrelsheikh University, Kafrelsheikh 33516, Egypt; 3 Department of Mathematics, Jamoum University College, Umm Al-Qura University, Jamoum, Makkah 21955, Saudi Arabia

**Keywords:** CTE, L–S, and G–N models, spherical hole, multi-phase-lag

## Abstract

This article introduces magneto-thermoelastic exchanges in an unbounded medium with a spherical cavity. A refined multi-time-derivative dual-phase-lag thermoelasticity model is applied for this reason. The surface of the spherical hole is considered traction-free and under both constant heating and external magnetic field. A generalized magneto-thermoelastic coupled solution is developed utilizing Laplace’s transform. The field variables are shown graphically and examined to demonstrate the impacts of the magnetic field, phase-lags, and other parameters on the field quantities. The present theory is examined to assess its validity including comparison with the existing literature.

## 1. Introduction

The thermoelastic responses of different structures with spherical cavities have received much attention because of their usefulness in many industrial applications. In the following, we restrict our attention to the application of continuums with spherical cavities. All the problems discussed are concerned with thermoelastic exchanges within the framework of several generalized thermoelasticity theories.

Generalized thermoelasticity models, with one or more relaxation times, have been proposed to modify the heat conduction equation. One of the original forms of the heat conduction equation, associated with gases theory, was introduced by Maxwell [1]. Another form was proposed within the framework of heat conduction in rigid structures by Cattaneo [2]. A third form was introduced by Dhaliwal and Sherief [3] by extension to the case of an anisotropic medium. To overcome the contradiction of an endless velocity of thermal waves intrinsic to classical coupled thermoelasticity (CTE) theory [4], attempts have been made by various investigators, for a range of reasons, to modify coupled thermoelasticity to entail a wave-type heat conduction equation.

Lord and Shulman (L–S) [5] developed generalized thermoelasticity theory presenting one relaxation time in Fourier’s law of heat conduction equation and therefore converting it into a hyperbolic type. Banerjee and Roychoudhuri [6] discussed the generalized theory of thermo-elasticity suggested by L–S [5] to examine thermo-visco-elastic wave propagation in an unlimited viscoelastic body of Kelvin–Voight type with a spherical hole. Sinha and Elsibai [7] discussed thermoelastic exchanges in an unlimited solid with a spherical inclusion considering L–S and G–L theories. Rakshit Kundu and Mukhopadhyay [8] described field variables in a viscoelastic body with a spherical hole. Youssef [9] described a problem of thermoelastic exchanges in a limitless body including a spherical hole subjected to a moving heat source according to L–S theory. Elhagary [10] described the problem of a thermoelastic unbounded solid including a spherical hole in the framework of L–S diffusion theory. Karmakar et al. [11] determined the temperatures, stress, displacement, and strain in an unbounded solid including a spherical hole in the framework of processes addressed by two-temperature theory (2TT).

Later, Green–Naghdi (G–N) [12,13,14] created three versions for generalized thermoelasticity that were identified as I, II, and III types. Mukhopadhyay [15,16] presented thermoelastic exchanges in an unbounded solid including a spherical hole in the framework of G–N theory. Mukhopadhyay and Kumar [17] considered thermoelastic exchanges in an infinite solid with a spherical hole in the framework of several theories. Allam et al. [18] investigated electro-magneto-thermoelastic exchanges in an infinite solid with a spherical hole in the framework of G–N theory. Banik and Kanoria [19] determined the thermoelastic quantities in an infinite solid with a spherical inclusion in the framework of the 2TT. Abbas [20] investigated a general solution to the field equations of 2TT in an unbounded medium with a spherical hole in the framework of the G–N model. Bera et al. [21] investigated the waves arising from the boundary of a spherical cavity in an infinite medium. Biswas [22] examined the thermoelastic exchange in a limitless body including a spherical cavity in the context of the G–N model. Chandrasekharaiah and Narasimha Murthy [23] considered thermoelastic exchanges in an infinite body including a spherical inclusion.

Green and Lindsay [24] pioneered an additional theory, known as the G–L model, that included two relaxation times. Roy Choudhuri and Chatterjee [25] studied spherically symmetric thermoelastic waves in an unbounded body containing a spherical hole. Sherief and Darwish [26] presented a problem of a thermoelastic unbounded solid containing a spherical hole in the framework of thermoelasticity theory with two relaxation times. Mukhopadhyay [27] discussed thermally induced vibrations of an unbounded viscoelastic body including a spherical hole in the framework of G–L theory. Ghosh and Kanoria [28] determined thermoelastic quantities in a functionally graded (FG) spherically unbonded body including a spherical hole in the framework of G–L theory. Kanoria and Ghosh [29] examined thermoelastic exchanges in an FG hollow sphere in the framework of the G–L model. Das and Lahiri [30] considered a thermoelastic problem for an unbounded FG and temperature-dependent spherical inclusion in the framework of G–L theory.

Many investigators have used dual/triple-phase-lag (D/TPL) heat transfer theory to examine thermoelastic exchanges in unbounded mediums including spherical cavities. DPL theory was originally presented by Tzou [31,32] to describe some problems at a macroscopic scale. Abouelregal and Abo-Dahab [33] presented thermal quantities in an unbounded solid with a spherical hole in the framework of DPL theory. Hobiny and Abbas [34] applied DPL theory in the examination of photo-thermal exchanges in an infinite solid containing a spherical cavity. Mondal and Sur [35] studied a coupled problem in an infinite solid with a spherical hole in the framework of a photothermal transport process in relation to 2TT. Singh and Sarkar [36] examined thermoelastic exchange in a 2TT unbounded isotropic body containing a spherical cavity in the framework of a memory-dependent derivative (MDD). Comparisons were made graphically between the 2T TPL theory and 2T L–S theory with MDD. Many researchers have dealt with one-dimensional (1D) problems in generalized thermoelasticity in unbounded mediums with spherical cavities [37,38,39,40,41,42,43,44].

In the current article, magneto-thermoelastic exchanges in an infinite solid with a spherical hole are studied with respect to multi-time-derivative thermoelasticity theories [45,46,47,48,49,50,51,52,53]. A refined DPL model is used for this purpose. The technique of Laplace transforms in the time domain is applied to obtain the governing equations analytically. The derived equations are solved and then Laplace inversion is carried out to obtain the field quantities numerically. For verification proposes, the outcomes are compared with those obtained previously. Additional results are presented graphically and others are reported for future comparison.

## 2. Basic Equations

**Figure 1:**
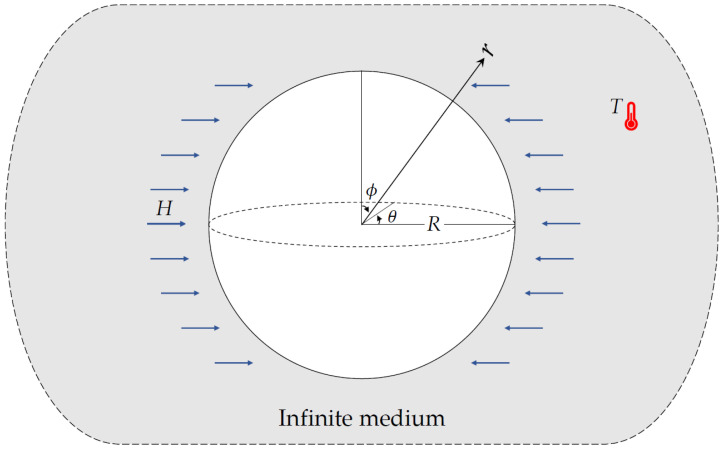
A spherical cavity in an unbounded medium under harmonically varying heat and external magnetic field.

Let us be concerned with thermoelastic analysis of an isotropic body including a spherical cavity of radius *R* based on unified multi-phase-lag theory. It is assumed that the outer edge of the spherical cavity is traction-free and subjected to harmonically varying heat (See Figure 1). The spherical cavity coordinate system (*r*,*θ*,*ϕ*) is used to address the present problem.

The governing equations for a linear isotropic homogeneous thermoelastic body in the absence of volume forces are given by:

- The equations of motion:

(1) $$\mu \;u_{i,jj}+\left(\lambda +\mu \right)u_{j,ij}-\gamma \Theta_{,i}=\rho \frac{\partial^{2}u_{i}}{\partial t^{2}}$$

- The constitutive equations:

(2) $$\sigma_{ij}=2\mu e_{ij}+\left(\lambda e_{k,k}-\gamma \Theta \right)\delta_{ij}$$

where $\sigma_{ij}$ and $e_{ij}$ are the stresses and strains and $\delta_{ij}$ denotes Kronecker’s delta tensor.

- The heat conduction equation:

(3) $$kL_{T}\left(\nabla^{2}\Theta \right)=L_{q}\left(\rho C_{e}\frac{\partial \Theta }{\partial t}+\gamma T_{0}\frac{\partial u_{k,k}}{\partial t}-Q\right)$$

is considered in the context of the refined thermoelasticity form in which $L_{T}$ and $L_{q}$ denote the following higher-order time-derivative operators:

(4) $$L_{T}=1+\overset{N}{\underset{n=1}{\sum }}\frac{\tau_{T}^{n}}{n!}\frac{\partial^{n}}{\partial t^{n}},\;\;\;\;\;L_{q}=\varrho +\overset{N}{\underset{n=1}{\sum }}\frac{\tau_{q}^{n}}{n!}\frac{\partial^{n}}{\partial t^{n}}$$

Equation (3) with the aid of Equation (2) are the more general ones when N has numerous integers more than zero. Some specific cases may be achieved as

(i) Dynamical coupled thermoelasticity (CTE) model [4]: $\tau_{T}=\tau_{q}=0$ and ϱ=1,
  (5) $$k\nabla^{2}\Theta =\rho C_{e}\frac{\partial \Theta }{\partial t}+\gamma T_{0}\frac{\partial u_{k,k}}{\partial t}-Q$$
(ii) Lord and Shulman (L–S) model [5]: $\tau_{T}=0$, $\tau_{q}=\tau_{0}$ and ϱ=1,
  (6) $$k\nabla^{2}\Theta =\left(1+\tau_{q}\frac{\partial }{\partial t}\right)\left(\rho C_{e}\frac{\partial \Theta }{\partial t}+\gamma T_{0}\frac{\partial u_{k,k}}{\partial t}-Q\right)$$
(iii) Green and Naghdi (G–N) model without energy dissipation [12,13,14]: $\tau_{T}=0$, $\tau_{q}=1$, $k\to k^{*}$, N=1 and ϱ=0,
  (7) $$k^{*}\nabla^{2}\Theta =\frac{\partial }{\partial t}\left(\rho C_{e}\frac{\partial \Theta }{\partial t}+\gamma T_{0}\frac{\partial u_{k,k}}{\partial t}-Q\right)$$
(iv) The simple dual-phase-lag (SDPL) model [50,51,52]: $\tau_{q}\geq \tau_{T}>0$, ϱ=1 and N=1,
  (8) $$k\left(1+\tau_{T}\frac{\partial }{\partial t}\right)\nabla^{2}\Theta =\left(1+\tau_{q}\frac{\partial }{\partial t}\right)\left(\rho C_{e}\frac{\partial \Theta }{\partial t}+\gamma T_{0}\frac{\partial u_{k,k}}{\partial t}-Q\right)$$
(v) The refined with dual-phase-lag (RDPL) model [50,51,52]: N>1, $\tau_{q}\geq \tau_{T}>0$, and ϱ=1,
  (9) $$k\left(1+\overset{N}{\underset{n=1}{\sum }}\frac{\tau_{T}^{n}}{n!}\frac{\partial^{n}}{\partial t^{n}}\right)\nabla^{2}\Theta =\left(1+\overset{N}{\underset{n=1}{\sum }}\frac{\tau_{q}^{n}}{n!}\frac{\partial^{n}}{\partial t^{n}}\right)\left(\rho C_{e}\frac{\partial \Theta }{\partial t}+\gamma T_{0}\frac{\partial u_{k,k}}{\partial t}-Q\right)$$

The displacements of the present, axially symmetric spherical medium are summarized as

(10) $$u_{r}=u\left(r,t\right),\;\;\;\;\;u_{\theta }=u_{\phi }=0$$

The non-vanishing strains and volumetric strain can be expressed as

(11) $$e_{rr}=\frac{\partial u}{\partial r},\;\;\;\;\;e_{\theta \theta }=e_{\phi \phi }=\frac{u}{r}$$

Thus, the volumetric strain e has the form

(12) $$e=e_{rr}+e_{\theta \theta }+e_{\phi \phi }=\frac{\partial u}{\partial r}+\frac{2u}{r}=\frac{1}{r^{2}}\frac{\partial }{\partial r}\left(r^{2}u\right)$$

The constitutive equations for the spherical symmetric system can be stated as

(13) $$\sigma_{rr}=2\mu \frac{\partial u}{\partial r}+\lambda e-\gamma \Theta$$

(14) $$\sigma_{\theta \theta }=\sigma_{\phi \phi }=2\mu \frac{u}{r}+\lambda e-\gamma \Theta$$

(15) $$\left(2\mu +\lambda +\mu_{0}H_{0}^{2}\right)\frac{\partial e}{\partial r}-\gamma \frac{\partial \Theta }{\partial r}=\rho \frac{\partial^{2}u}{\partial t^{2}}$$

Applying the operator (∂/∂r+2/r) to both sides of Equation (15), one gets

(16) $$\left(2\mu +\lambda +\mu_{0}H_{0}^{2}\right)\nabla^{2}e-\gamma \nabla^{2}\Theta =\rho \frac{\partial^{2}e}{\partial t^{2}}$$

in which $\nabla^{2}$ denotes the Laplacian operator in spherical coordinates. It meets the formulation

(17) $$\nabla^{2}\left(*\right)=\frac{\partial^{2}\left(*\right)}{\partial r^{2}}+\frac{2}{r}\frac{\partial \left(*\right)}{\partial r}=\frac{1}{r^{2}}\frac{\partial }{\partial r}\left(r^{2}\frac{\partial \left(*\right)}{\partial r}\right)$$

## 3. Formulation of the Problem

It is proper to establish the non-dimensional variables in the following parts:

(18) $$\begin{array}{c} \left\{r^{\prime },u^{\prime }\right\}=c_{0}\eta \left\{r,u\right\},\left\{t^{\prime },\tau_{T}^{\prime },\tau_{q}^{\prime }\right\}=\eta c_{0}^{2}\left\{t,\tau_{T},\tau_{q}\right\},\\ \sigma_{ii}^{\prime }=\frac{\sigma_{ii}}{\lambda +2\mu },\;\;\;\;\;\Theta^{\prime }=\frac{\gamma \Theta }{\lambda +2\mu },\;\;\;\;\;c_{0}^{2}=\frac{\lambda +2\mu }{\rho },\;\;\;\;\;\eta =\frac{\rho C_{e}}{k} \end{array}$$

The whole governing equations, with the above dimensionless variables, are diminished to (throwing down the dash for convenience)

(19) $$\sigma_{rr}=e-\frac{2}{c_{1}}\frac{u}{r}-\Theta$$

(20) $$\sigma_{\theta \theta }=\sigma_{\phi \phi }=\left(1-\frac{1}{c_{1}}\right)e+\frac{1}{c_{1}}\frac{u}{r}-\Theta$$

(21) $$c_{2}\nabla^{2}e-\nabla^{2}\Theta =\frac{\partial^{2}e}{\partial t^{2}}$$

(22) $$\left(\nabla^{2}L_{T}-L_{q}\frac{\partial }{\partial t}\right)\Theta -\varepsilon L_{q}\left(\frac{\partial e}{\partial t}\right)=0$$

where

(23) $$c_{1}=\frac{\lambda +2\mu }{2\mu },\;\;\;\;\;c_{2}=1+\frac{\mu_{0}H_{0}^{2}}{\lambda +2\mu },\;\;\;\;\;\varepsilon =\frac{\gamma^{2}T_{0}}{\rho C_{e}\left(\lambda +2\mu \right)}$$

## 4. Closed-Form Solution

The comprehensive solutions are provided by resolving Equations (21) and (22) to obtain, firstly, temperature Θ and volumetric strain (dilatation) e. Then, the subsequent radial displacement and thermal stresses may be presented as functions of Θ and e. For this objective, we will first employ the next initial conditions:

(24) $$u\left(r,0\right)={\frac{\partial u}{\partial t}|}_{t=0}=0,\;\;\;\;\;\Theta \left(r,0\right)={\frac{\partial \Theta }{\partial t}|}_{t=0}=0,\;\;\;\;\;\;R\leq r<\infty$$

In adding together to the above homogenous initial conditions, we also used the thermomechanical boundary conditions. The current unbounded body will be studied as quiescent and the surface of the spherical cavity is assumed to be exposed to constant heat and traction free. Such conditions can be explained as

- The surface of the spherical hole is subjected to a constant heat

(25) $$\Theta \left(R,t\right)=\Theta_{0}H\left(t\right),\;\;\;\;\;\;t>0$$

- The mechanical boundary condition is respected as the surface of the spherical hole is traction free

(26) $$\sigma_{rr}\left(R,t\right)=0,\;\;\;\;\;\;t>0$$

Moreover, we take into consideration the following regularity conditions

(27) u(r,t)=0,     Θ(r,t)=0,      r→∞

The Laplace transform is carried out for Equations (19)–(22), and, with the homogeneous initial conditions that appeared in Equation (24), one gets:

(28) $${\bar{\sigma }}_{rr}=\bar{e}-\frac{2}{c_{1}}\frac{\bar{u}}{r}-\bar{\Theta }$$

(29) $${\bar{\sigma }}_{\theta \theta }={\bar{\sigma }}_{\phi \phi }=\left(1-\frac{1}{c_{1}}\right)\bar{e}+\frac{1}{c_{1}}\frac{\bar{u}}{r}-\bar{\Theta }$$

(30) $$\left(c_{2}\nabla^{2}-s^{2}\right)\bar{e}-\nabla^{2}\bar{\Theta }=0$$

(31) $$\left(\nabla^{2}-\varpi \right)\bar{\Theta }-\varepsilon \varpi \bar{e}=0$$

where

(32) $$\varpi =\frac{s{\bar{L}}_{q}}{{\bar{L}}_{T}},\;\;\;\;\;{\bar{L}}_{T}=1+\overset{N}{\underset{n=1}{\sum }}\frac{\tau_{T}^{n}}{n!}s^{n},\;\;\;\;\;{\bar{L}}_{q}=\varrho +\overset{N}{\underset{n=1}{\sum }}\frac{\tau_{q}^{n}}{n!}s^{n}$$

The system of equations provided in Equations (30) and (31) can be indicated in the differential equation

(33) $$\left(\nabla^{4}-\beta_{1}\nabla^{2}+\beta_{0}\right)\bar{e}\left(r\right)=0$$

where the coefficients $\beta_{i}$ are given by

(34) $$\beta_{0}=\frac{s^{2}\varpi }{c_{2}},\;\;\;\;\;\beta_{1}=\frac{s^{2}+\varpi \left(\varepsilon +c_{2}\right)}{c_{2}}$$

and the temperature $\bar{\Theta }$ is reformed as follows

(35) $$\bar{\Theta }\left(r\right)=\frac{c_{2}}{\varpi }\nabla^{2}\bar{e}\left(r\right)-\left(\frac{s^{2}}{\varpi }+\varepsilon \right)\bar{e}\left(r\right)$$

Equation (33) is very complicated since it is presented in a polar coordinate system. It can be expressed as

(36) $$\left(\nabla^{2}-\zeta_{1}^{2}\right)\left(\nabla^{2}-\zeta_{2}^{2}\right)\;\bar{e}\left(r\right)=0$$

where $\zeta_{j}^{2}$ are the roots of

(37) $$\zeta^{4}-\beta_{1}\zeta^{2}+\beta_{0}=0$$

These roots $\zeta_{j}$ are given, respectively, by

(38) $$\zeta_{1,2}^{2}=\frac{1}{2}\left(\beta_{1}\pm \sqrt{\beta_{1}^{2}-4\beta_{0}}\right)$$

Equation (36) tends to the next modified Bessel’s equation of zero-order

(39) $$\left(\frac{1}{r}\frac{\partial }{\partial r}\left(r\frac{\partial }{\partial r}\right)-\zeta_{1}^{2}\right)\left(\frac{1}{r}\frac{\partial }{\partial r}\left(r\frac{\partial }{\partial r}\right)-\zeta_{2}^{2}\right)\bar{e}\left(r\right)=0$$

which has a solution under the regularity conditions: $\bar{u}$, $\bar{\Theta }\to 0$ as r→∞. Therefore, the general solution of Equations (35) and (39), that is bounded at infinity, is provided by

(40) $$\left\{\bar{\Theta }\left(r\right),\bar{e}\left(r\right)\right\}=\frac{1}{r}\overset{2}{\underset{j=1}{\sum }}\left\{1,\zeta_{j}\right\}B_{j}e^{-\zeta_{j}r}$$

where $B_{j}$ are integration parameters and

(41) $$\zeta_{j}=\frac{c_{2}\xi_{2}^{2}-s^{2}}{\varpi }-\varepsilon$$

Using the relation between $\bar{u}$ and $\bar{e}$

(42) $$\bar{e}\left(r\right)=D\bar{u}\left(r\right),\;\;\;\;D=\frac{d}{dr}+\frac{2}{r}$$

one can pick up the solution for the dimensionless form of radial displacement pretending that $\bar{u}$ disappears at infinity as:

(43) $$\bar{u}\left(r\right)=\overset{2}{\underset{j=1}{\sum }}\left(1-{\hat{\zeta }}_{j}\right)B_{j}e^{-\zeta_{j}r}$$

where

(44) $${\hat{\zeta }}_{j}=1+\frac{1}{\zeta_{j}r}+\frac{1}{\zeta_{j}^{2}r^{2}\;}$$

Up to here, the solution is finished. It is as much as needed to establish the two parameters $B_{j}$ with the aid of the boundary conditions given in Equations (25) and (26). So, one gets

(45) $${\bar{\sigma }}_{1}={\bar{\sigma }}_{rr}=\frac{1}{r}\overset{2}{\underset{j=1}{\sum }}\left[1-\zeta_{j}+\frac{2}{c_{1}}\left({\hat{\zeta }}_{j}-1\right)\right]B_{j}e^{-\zeta_{j}r}$$

(46) $${\bar{\sigma }}_{2}={\bar{\sigma }}_{\theta \theta }={\bar{\sigma }}_{\phi \phi }=\frac{1}{r}\overset{2}{\underset{j=1}{\sum }}\left(1-\frac{{\hat{\zeta }}_{j}}{c_{1}}-\zeta_{j}\right)B_{j}e^{-\zeta_{j}r}$$

Therefore, the current analytical solution is already provided for the modified formulations in Laplace space. To achieve the solution in the basic time-space one can consider a function ψ(t) as an inversion of the Laplace function $\bar{\psi }\left(s\right)$ in the form

(47) ψ(t)=eptt[12ψ¯(p)+Re(∑ι=1L(−1)ιψ¯(p+iιπt)) ]

where p is an arbitrary constant, Re is the real part, i suggests the imagined number unit and L denotes a sufficiently big integer. For faster combination, various numerical analyses have shown that the approximation of p fulfills the connection pt≈4.7 [35]. The numerical procedure cited is used to invert the terms of temperature Θ, radial displacement u, volumetric strain e, radial stress $\sigma_{1}$, and circumferential stress $\sigma_{2}$.

## 5. Validation

Numerous examples are presented to illustrate the effect of several models on the field variables. The material properties of the infinite medium with a spherical cavity are

$$\lambda =7.76\times 10^{10}{\;N\;m}^{-2},\;\mu =3.86\times 10^{10}{\;N\;m}^{-2},\;C_{e}=383.1{\;J\;\mathrm{kg}}^{-1}{\;K}^{-1},\;\alpha_{t}=1.78\times 10^{-5}{\;K}^{-1},\;\rho =8954{\;\mathrm{kg}\;m}^{-3},\;k=386{\;W\;m}^{-1}{\;K}^{-1},\;T_{0}=293\;K,\;k^{*}=1.2$$

Numerical outcomes are attained (except where otherwise indicated) for $\Theta_{0}=10$, $\tau_{q}=0.02$, $\tau_{T}=0.018$, t=0.03, and radius R=1.

### 5.1. First Justification

**Figure 2:**
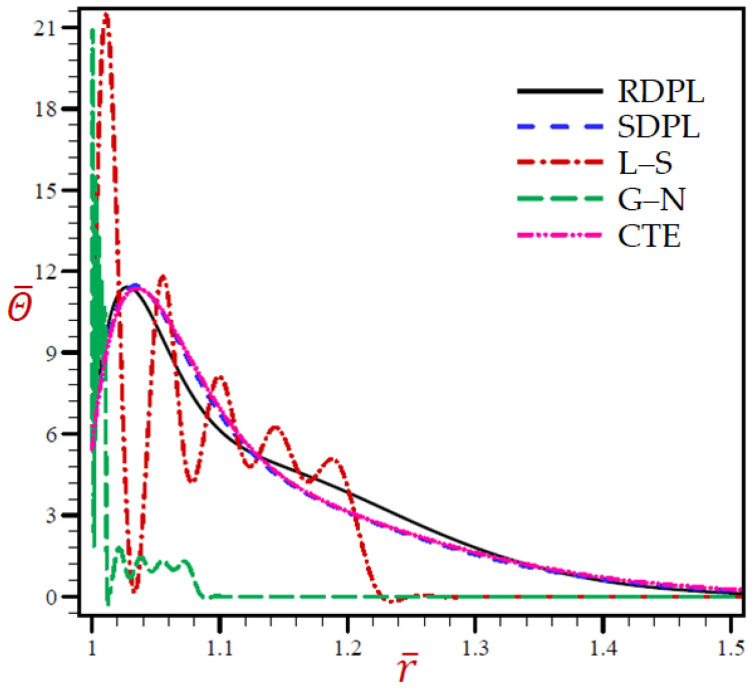
The temperature $\bar{\Theta }$ through radial direction of spherical hole presenting to all models.

**Figure 3:**
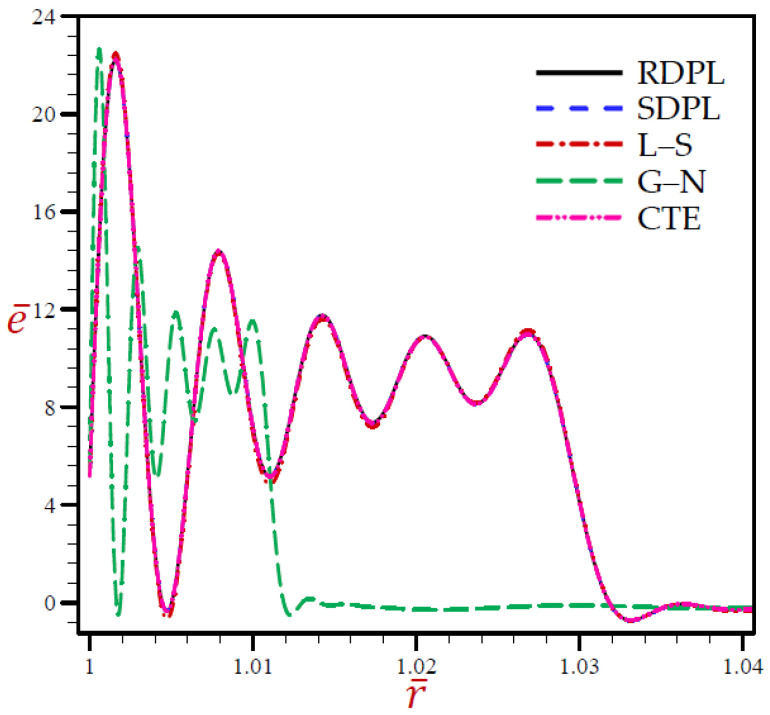
The volumetric strain $\bar{e}$ through radial direction of a spherical hole presenting to all models.

**Figure 4:**
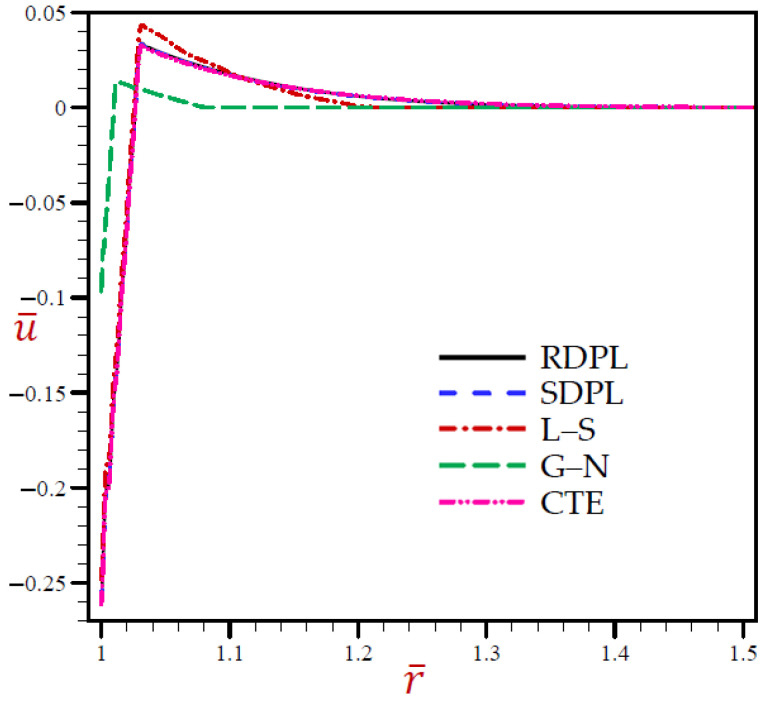
The radial displacement $\bar{u}$ through radial direction of spherical hole presenting to all models.

**Figure 5:**
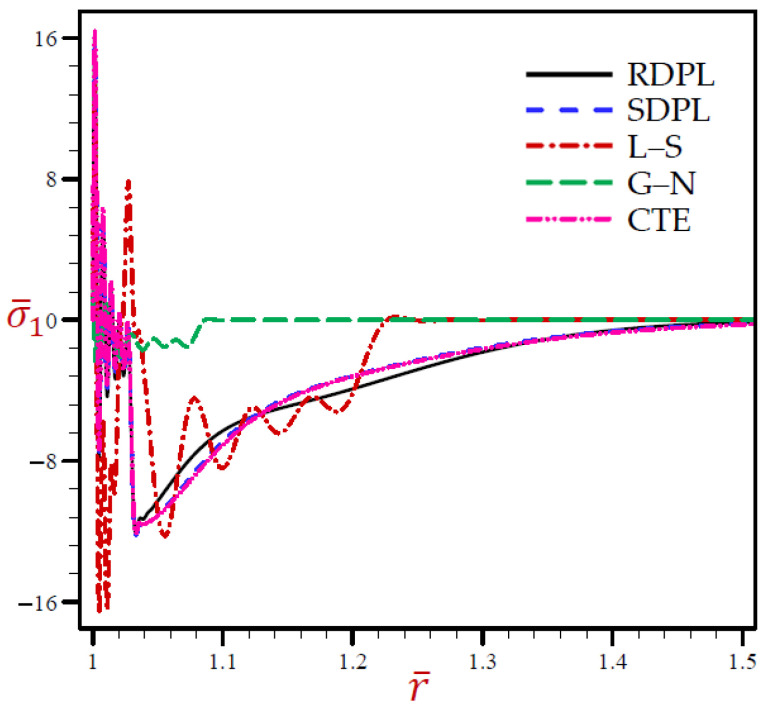
The radial stress ${\bar{\sigma }}_{1}$ through radial direction of spherical hole presenting to all models.

**Figure 6:**
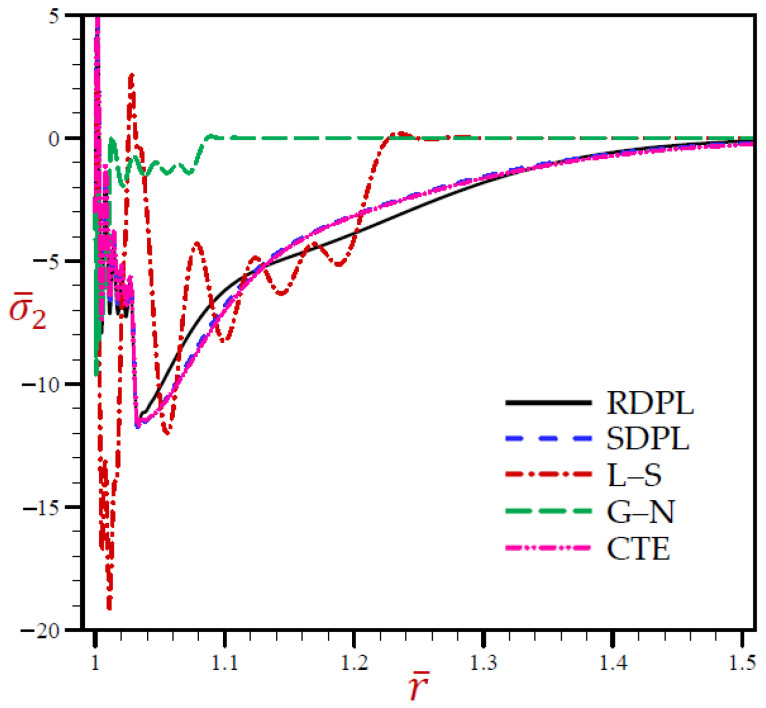
The circumferential stress ${\bar{\sigma }}_{2}$ through radial direction of a spherical hole presenting to all models.

**Figure 7:**
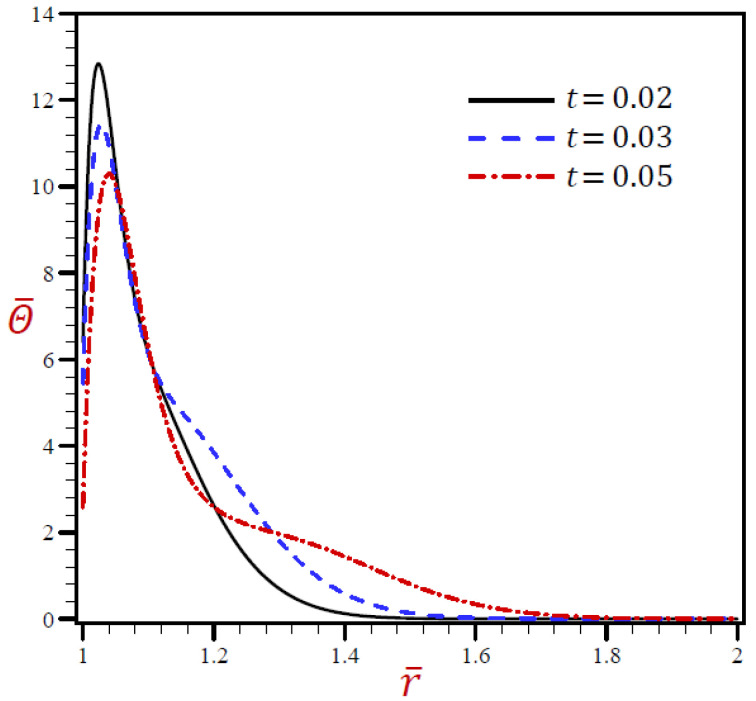
The influence of *t* on temperature $\bar{\Theta }$ through radial direction of a spherical hole using RDPL model.

**Figure 8:**
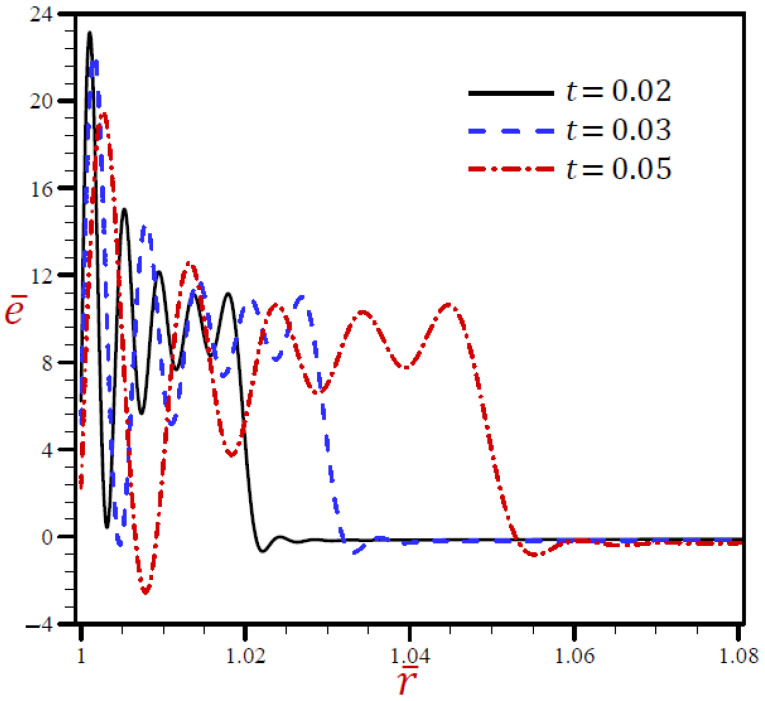
The influence of t on volumetric strain $\bar{e}$ through radial direction of spherical hole using RDPL model.

**Figure 9:**
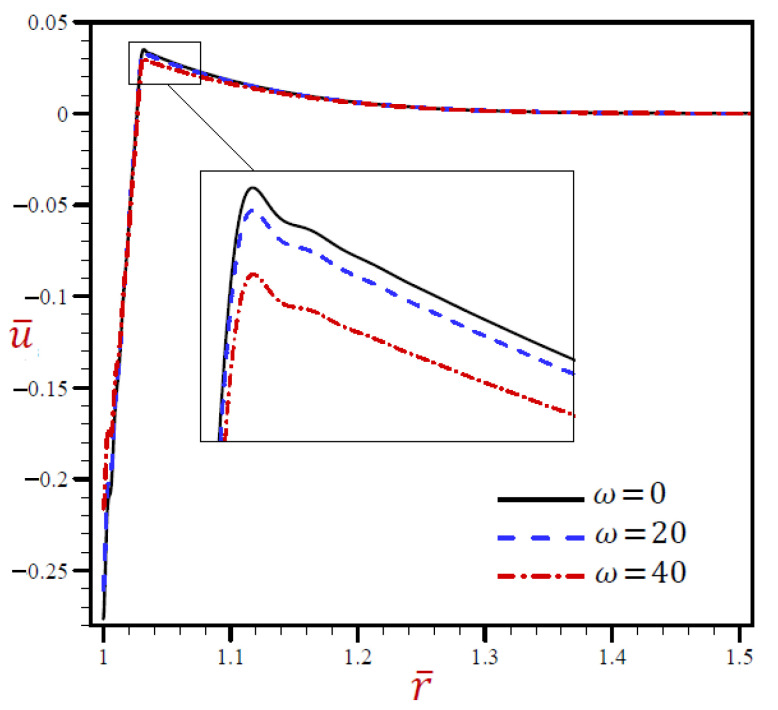
The influence of t on radial displacement $\bar{u}$ through radial direction of spherical hole using RDPL model.

**Figure 10:**
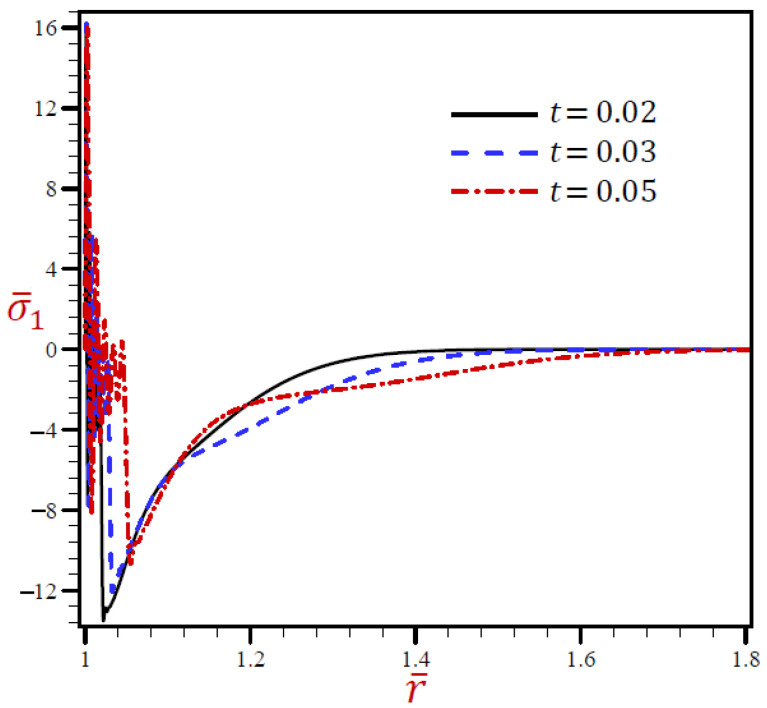
The influence of t on radial stress ${\bar{\sigma }}_{1}$ through radial direction of spherical hole using RDPL model.

**Figure 11:**
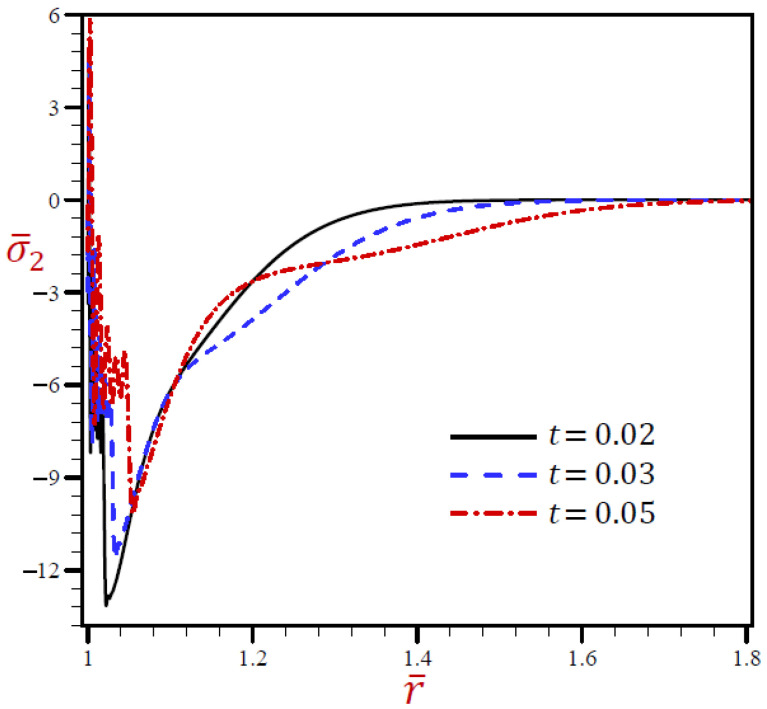
The influence of t on circumferential stress ${\bar{\sigma }}_{2}$ through radial direction of spherical hole using RDPL model.

The outcomes for all variables using various thermoelasticity models of dual-phase-lag are presented in Table 1, Table 2, Table 3, Table 4 and Table 5 at different positions. The impact of magnetic field $\mu_{0}$ and $H_{0}$ on all field quantities of the various models are produced at dimensionless time t=0.03. Additional results are illustrated in Figure 2, Figure 3, Figure 4, Figure 5, Figure 6, Figure 7, Figure 8, Figure 9, Figure 10 and Figure 11 through the radial direction of an unbounded medium with a spherical hole.

The outcomes described in Table 1, Table 2, Table 3, Table 4 and Table 5 are offered as benchmarks for other researchers. It is evident from the tabulated results that:

- The G–N model provides the lowest absolute value of all variables. It may vanish at some positions.
- The other CTE and L–S models provide appropriate outcomes for all variables.
- Triplet values *N* = 3, 4, and 5 are utilized for the RDPL model while the SDPL model is defined with *N* = 1.
- Extremely exact outcomes are provided utilizing the RDPL model.
- For the RDPL model the temperature, displacement, and circumferential stress slightly increase as the value of *N* increases, while volumetric strain, radial stress, and circumferential stress slightly decrease. All variables may be insensitive to the higher values of *N* especially when *N* ≥ 5.

### 5.2. Second Justification

Figure 2, Figure 3, Figure 4, Figure 5 and Figure 6 show the effect of all models on the variables with fixed time *t* = 0.03. The remainder of the graphs are exhibited in relation to the refined dual-phase-lag (RDPL) model with *N* = 5 to examine the effect of various parameters on all field variables.

The discrepancy of the temperature $\bar{\Theta }$ through radial direction of a spherical hole corresponding to all models is produced in Figure 2. Similar figures for the remaining variables are presented in Figure 3, Figure 4, Figure 5 and Figure 6. Figure 2 reveals that the temperature due to the CTE, L–S, and SDPL models vibrates across the trajectory of the RDPL model, while temperature due to the G–N model vibrates below the trajectory of the RDPL model. The temperature according to the G–N model may vanish earlier than the temperature according to other models.

Figure 3 reveals that the values of $\bar{e}$ of SDPL, L–S, and CTE models vibrate identically to the trajectory of the RDPL theory. While the value of $\bar{e}$ for the G–N model vibrates across and below the trajectory of the RDPL model.

Figure 4 indicates that the radial displacements $\bar{u}$ of the CTE and SDPL models may be the same as those of RDPL theory, vanishing through radial direction. The displacements $\bar{u}$ of the L–S model may be the upper or lower bounds of those due to the RDPL model. The G–N model always produces the smallest displacement during the radial direction.

Figure 5 reveals that the radial stress ${\bar{\sigma }}_{1}$ of the G–N model may rapidly vanish through the radial direction when *r* > 1.1. The radial stress of the L–S model vibrates around the RDPL model with wide amplitude, then it also vanishes when *r* > 1.24. The other CTE and SDPL theories give radial stresses that vibrate around those of the RDPL theory but with small amplitude.

Finally, Figure 6 shows similar behaviors of circumferential stress as those of the radial stress. It shows that the circumferential stress ${\bar{\sigma }}_{2}$ of the G–N model may rapidly vanish through the radial direction when *r* > 1.1. The radial stress of the L–S model vibrates around the RDPL theory with wide amplitude, then it also vanishes when *r* > 1.24. The other CTE and SDPL theories give radial stresses that vibrate around those of the RDPL theory but with small amplitude.

It is concluded from the above figures that the outcomes of the RDPL model are the most straightforward. So, we restrict our attention to using this theory for yielding the outcomes of this problem considering the effect of various parameters on the field variables.

### 5.3. The Influence of Dimensionless Time

The outcomes of dimensionless time t on all variables due to the RDPL model are presented in Figure 7, Figure 8, Figure 9, Figure 10 and Figure 11. Figure 7 reveals the effects of *t* on $\bar{\Theta }$ through radial direction of a spherical hole. Similar figures for the remaining variables are presented in Figure 8, Figure 9, Figure 10 and Figure 11. It is clear in Figure 7 that $\bar{\Theta }$ vibrates through the radial direction for various values of *t* with different wavelengths. The temperature $\bar{\Theta }$ no longer increases and has its highest values when *r* = 1.04. The temperature vanishes as *r* increases, irrespective of the values of *t*.

Figure 8 reveals that the volumetric strain $\bar{e}$ vibrates through the radial direction of a spherical hole with different amplitudes and different wavelengths. The wavelength increases as t increases. For t=0.02 the volumetric strain $\bar{e}$ firstly vanishes when r>1.024, while for t=0.05, the volumetric strain $\bar{e}$ finally vanishes when r>1.06. In Figure 9, the radial displacement $\bar{u}$ rapidly increases through the radial direction of the spherical hole when t=0.02, while $\bar{u}$ slowly increases when t=0.03. $\bar{u}$ is slowly decreasing when t=0.05 It is obvious that the radial displacement $\bar{u}$ increases with increase in dimensionless time t at fixed positions.

The radial stress ${\bar{\sigma }}_{1}$ through the radial direction of spherical hole due to the RDPL model is described in Figure 10 for various values of t. The radial stress ${\bar{\sigma }}_{1}$ oscillates on a very small scale, then increases when t=0.03 and 0.05, while it decreases when t=0.02. At any fixed position, the radial stress ${\bar{\sigma }}_{1}$ increases with increase in the dimensionless time t. The circumferential stress ${\bar{\sigma }}_{2}$ is drawn through the radial direction of the spherical cavity utilizing the RDPL model in Figure 11 for distinctive values of t. It vibrates over a very small range, then it increases for t=0.02, but decreases when t=0.03 and 0.05. At any fixed position, the circumferential stress ${\bar{\sigma }}_{2}$ increases with increase in the dimensionless time t.

## 6. Conclusions

The present refined dual-phase-lag model is innovative and produces accurate results for variables such as temperature, volumetric strain, displacement, and stresses. The multi-time derivatives heat equation was explained. The constitutive relations of spherical coordinates were considered to examine the thermoelastic coupling behavior of an infinite medium with a spherical cavity due to a uniform heat. To create a unified model, one can combine other models, including the coupled dynamical thermoelasticity model, the Lord and Shulman model, the Green and Naghdi model without energy dissipation, as well as a simple dual-phase-lag model. The system of two high-time-derivative differential coupled equations was solved, and all field variables were developed for the thermoelastic coupling response of an infinite medium with a spherical hole. Various confirmation examples and applications were offered to compare the outcomes due to all models with the refined ones. A sample set of graphs were presented to demonstrate relationships of variables through radial direction of a spherical hole. Some tables have been provided as confirmation examples to provide benchmark outcomes for future comparisons by other researchers. The described and demonstrated outcomes revealed different behaviors of all field variables and dimensionless time parameters. The present dual-phase-lag theory diminished the magnitudes of the examined variables, which may be significant in some practical applications. The G–N model provided appropriate outcomes over a small range. However, the refined model produced improved and exact outcomes.

## Figures and Tables

**Table 1:** Effects of dimensionless time *t* on volumetric strain $\bar{e}$ according to different thermoelasticity theories with several values of *r*.

*r*	*t*	CTE	G–N	L–S	SDPL	RDPL		
					*N* = 1	*N* = 3	*N* = 4	*N* = 5
1.001	0.02	23.809257	3.0601024	24.0856	23.808792	23.808531	23.808844	23.809054
	0.03	21.299459	16.83015	21.555949	21.29979	21.30039	21.299517	21.295514
	0.05	16.62998	24.126713	16.819316	16.631298	16.631497	16.630909	16.636946
1.0108	0.02	9.048562	−0.1153723	8.8444984	9.0436695	9.0430854	9.0508697	9.0662687
	0.03	5.9635525	7.3662102	5.6387135	5.9605812	5.9665798	5.9730733	5.9683018
	0.05	9.6360547	7.6797451	9.459863	9.6357821	9.6349646	9.6210413	9.6310639
1.035	0.02	−0.1538211	−0.2026603	−0.4084073	−0.1628925	−0.1754834	−0.1766143	−0.1722681
	0.03	−0.2531288	−0.2064843	−0.2693186	−0.2655987	−0.2744405	−0.2676814	−0.2565349
	0.05	10.563634	−0.3048223	10.574561	10.558	10.573683	10.573157	10.549094

**Table 2:** Effects of dimensionless time *t* on radial displacement $\bar{u}$ according to different thermoelasticity theories with several values of *r*.

*r*	*t*	CTE	G–N	L–S	SDPL	RDPL		
					*N* = 1	*N* = 3	*N* = 4	*N* = 5
1.02	0.02	0.016967	6.59 × 10^−3^	0.02626	0.017492	0.018306	0.018521	0.018544
	0.03	−0.06242	0.012333	−0.05158	−0.06156	−0.06069	−0.06086	−0.06142
	0.05	−0.21674	0.023884	−0.20169	−0.21541	−0.21513	−0.21532	−0.2143
1.2	0.02	2.17 × 10^−3^	6.68 × 10^−11^	3.38 × 10^−6^	2.04 × 10^−3^	1.82 × 10^−3^	1.73 × 10^−3^	1.68 × 10^−3^
	0.03	6.32 × 10^−3^	5.89 × 10^−7^	9.67 × 10^−4^	6.12 × 10^−3^	5.89 × 10^−3^	5.91 × 10^−3^	6.05 × 10^−3^
	0.05	0.02141	1.46 × 10^−6^	0.018332	0.021199	0.021304	0.021448	0.021156
1.4	0.02	1.09 × 10^−4^	8.19 × 10^−19^	7.92 × 10^−8^	8.52 × 10^−5^	5.03 × 10^−5^	3.80 × 10^−5^	2.83 × 10^−5^
	0.03	6.20 × 10^−4^	3.73 × 10^−12^	1.20 × 10^−6^	5.33 × 10^−4^	4.03 × 10^−4^	3.58 × 10^−4^	3.26 × 10^−4^
	0.05	3.81 × 10^−3^	4.67 × 10^−8^	8.92 × 10^−6^	3.54 × 10^−3^	3.30 × 10^−3^	3.34 × 10^−3^	3.47 × 10^−3^

**Table 3:** Effects of dimensionless time *t* on temperature $\bar{\Theta }$ according to different thermoelasticity theories with several values of *r*.

*r*	*t*	CTE	G–N	L–S	SDPL	RDPL		
					*N* = 1	*N* = 3	*N* = 4	*N* = 5
1.02	0.02	12.4075	0.765094	2.199177	12.55818	12.92892	13.1502	13.34968
	0.03	11.96567	1.840952	14.22474	12.14601	12.5459	12.66462	12.52622
	0.05	11.31646	0.063337	22.12826	11.50767	11.8686	12.0525	12.76183
1.2	0.02	2.604722	−2.25 × 10^−8^	0.014782	2.510894	2.453168	2.515533	2.661198
	0.03	3.361241	1.08 × 10^−4^	4.068197	3.310861	3.477755	3.716377	4.020897
	0.05	4.423428	−1.49 × 10^−4^	4.010311	4.357168	4.381843	4.120538	3.461431
1.4	0.02	0.325147	1.79 × 10^−16^	1.45 × 10^−4^	0.268041	0.181612	0.14935	0.122447
	0.03	0.733066	5.64 × 10^−9^	4.00 × 10^−4^	0.660726	0.580455	0.574048	0.591092
	0.05	1.471241	5.49 × 10^−5^	0.06287	1.423396	1.488998	1.566341	1.558591

**Table 4:** Effects of dimensionless time t on radial stress ${\bar{\sigma }}_{1}$ according to different thermoelasticity theories with several values of r.

*r*	*t*	CTE	G–N	L–S	SDPL	RDPL		
					*N* = 1	*N* = 3	*N* = 4	*N* = 5
1.02	0.02	−8.18367	−0.89255	2.057934	−8.34605	−8.73216	−8.95355	−9.14535
	0.03	−1.07491	−2.14559	−3.37834	−1.26233	−1.65585	−1.75897	−1.60453
	0.05	−4.01791	−0.38448	−15.1083	−4.2121	−4.56849	−4.77181	−5.5112
1.2	0.02	−2.6318	2.61 × 10^−8^	−0.01511	−2.53776	−2.47972	−2.54218	−2.68827
	0.03	−3.42287	−1.26 × 10^−4^	−4.15875	−3.37323	−3.54214	−3.78243	−4.08913
	0.05	−4.57844	1.71 × 10^−4^	−4.20834	−4.51548	−4.54388	−4.28088	−3.61623
1.4	0.02	−0.32688	−2.07 × 10^−15^	−1.48 × 10^−4^	−0.2695	−0.1826	−0.15016	−0.1231
	0.03	−0.74048	−6.54 × 10^−9^	−4.15 × 10^−4^	−0.6675	−0.58625	−0.57954	−0.59643
	0.05	−1.50361	−6.36 × 10^−5^	0.064107	−1.45493	−1.52053	−1.59878	−1.59192

**Table 5:** Effects of dimensionless time t on circumferential stress ${\bar{\sigma }}_{2}$ according to different thermoelasticity theories with several values of r.

*r*	*t*	CTE	G–N	L–S	SDPL	RDPL		
					*N* = 1	*N* = 3	*N* = 4	*N* = 5
1.02	0.02	−10.2735	−0.82254	−0.03941	−10.4295	−10.8072	−11.0283	−11.2239
	0.03	−6.56734	−1.98159	−8.83803	−6.75038	−7.14623	−7.2573	−7.1114
	0.05	−7.86997	−0.20094	−18.8067	−8.06137	−8.41975	−8.61358	−9.33697
1.2	0.02	−2.61649	2.44 × 10^−8^	−0.01494	−2.52266	−2.46497	−2.52745	−2.67337
	0.03	−3.38687	−1.17 × 10^−4^	−4.11278	−3.33703	−3.50513	−3.74457	−4.05007
	0.05	−4.48332	1.61 × 10^−4^	−4.09433	−4.41888	−4.44534	−4.18307	−3.52142
1.4	0.02	−0.32594	−1.93 × 10^−15^	−1.46 × 10^−4^	−0.26871	−0.18207	−0.14973	−0.12275
	0.03	−0.73634	−6.09 × 10^−9^	−4.07 × 10^−4^	−0.66374	−0.58307	−0.57655	−0.59354
	0.05	−1.48475	−5.92 × 10^−5^	0.063495	−1.43668	−1.50245	−1.58022	−1.57282

## Data Availability

Not applicable.

## Nomenclature

$\alpha_{t}$	thermal expansion $\left(K^{-1}\right)$
$C_{\vartheta }$	specific heat $\left(J\;{\mathrm{kg}}^{-1}\;K^{-1}\right)$
$\delta_{ij}$	Kronecker’s delta
$e_{\theta \theta },e_{\phi \phi }$	circumferential strains
$e_{rr}$	radial strain
e	volumetric strain (dilatation)
$e_{ij}$	strain tensor components
ϑ	velocity of heat source $\left({m\;s}^{-1}\right)$
$\gamma \equiv \left(3\lambda +2\mu \right)\alpha_{t}$	thermal modulus $\left({N\;m}^{-2}{\;K}^{-1}\right)$
H(t)	Heaviside’s unit step function
$H_{0}$	initial magnetic field
k	heat conductivity $\left({W\;m}^{-1}{\;K}^{-1}\right)$
$k^{*}$	rate of thermal conductivity $\left({W\;m}^{-1}{\;K}^{-1}\right)$
λ,μ	Lame’s constants $\left({N\;m}^{-2}\right)$
$\mu_{0}$	electric permeability
ρ	density $\left({\mathrm{kg}\;m}^{-3}\right)$
R	radius of the spherical hole (m)
(r,θ,ϕ)	spherical coordinates system
$\sigma_{ij}$	stress tensor components $\left({N\;m}^{-2}\right)$
$\sigma_{\theta \phi },\sigma_{r\theta },\sigma_{r\phi }$	shear stresses $\left({N\;m}^{-2}\right)$
$\sigma_{\theta \theta },\sigma_{\phi \phi }$	circumferential stresses $\left({N\;m}^{-2}\right)$
$\sigma_{rr}$	radial stress $\left({N\;m}^{-2}\right)$
s	Laplace parameter
$\Theta =T-T_{0}$	temperature change (K)
$\Theta_{0}$	thermal constant (K)
$T_{0}$	environment temperature (K)
$\tau_{q}$	phase-lag of heat flux (s)
$\tau_{T}$	phase-lag of temperature gradient (s)
$\tau_{0}$	first relaxation time (s)
ω	angular frequency of thermal vibration $\left({\mathrm{rad}\;s}^{-1}\right)$
$Q_{0}$	strength of heat source $\left({W\;m}^{-3}\right)$
δ	delta function
$\vec{q}$	heat flux vector $\left({W\;m}^{-2}\right)$
$u_{r}$	radial displacement (m)
$u_{\theta },u_{\phi }$	circumferential displacements (m)
